# Applying probability calibration to ensemble methods to predict 2-year mortality in patients with DLBCL

**DOI:** 10.1186/s12911-020-01354-0

**Published:** 2021-01-07

**Authors:** Shuanglong Fan, Zhiqiang Zhao, Hongmei Yu, Lei Wang, Chuchu Zheng, Xueqian Huang, Zhenhuan Yang, Meng Xing, Qing Lu, Yanhong Luo

**Affiliations:** 1 Department of Health Statistics, School of Public Health, Shanxi Medical University, Taiyuan, China; 2 Department of Hematology, Shanxi Cancer Hospital, Taiyuan, China; 3 Department of Epidemiology and Biostatistics, Michigan State University, East Lansing, USA

**Keywords:** DLBCL, Risk prediction, Probability calibration, Ensemble method, Discrimination, Calibration

## Abstract

**Background:**

Under the influences of chemotherapy regimens, clinical staging, immunologic expressions and other factors, the survival rates of patients with diffuse large B-cell lymphoma (DLBCL) are different. The accurate prediction of mortality hazards is key to precision medicine, which can help clinicians make optimal therapeutic decisions to extend the survival times of individual patients with DLBCL. Thus, we have developed a predictive model to predict the mortality hazard of DLBCL patients within 2 years of treatment.

**Methods:**

We evaluated 406 patients with DLBCL and collected 17 variables from each patient. The predictive variables were selected by the Cox model, the logistic model and the random forest algorithm. Five classifiers were chosen as the base models for ensemble learning: the naïve Bayes, logistic regression, random forest, support vector machine and feedforward neural network models. We first calibrated the biased outputs from the five base models by using probability calibration methods (including shape-restricted polynomial regression, Platt scaling and isotonic regression). Then, we aggregated the outputs from the various base models to predict the 2-year mortality of DLBCL patients by using three strategies (stacking, simple averaging and weighted averaging). Finally, we assessed model performance over 300 hold-out tests.

**Results:**

Gender, stage, IPI, KPS and rituximab were significant factors for predicting the deaths of DLBCL patients within 2 years of treatment. The stacking model that first calibrated the base model by shape-restricted polynomial regression performed best (AUC = 0.820, ECE = 8.983, MCE = 21.265) in all methods. In contrast, the performance of the stacking model without undergoing probability calibration is inferior (AUC = 0.806, ECE = 9.866, MCE = 24.850). In the simple averaging model and weighted averaging model, the prediction error of the ensemble model also decreased with probability calibration.

**Conclusions:**

Among all the methods compared, the proposed model has the lowest prediction error when predicting the 2-year mortality of DLBCL patients. These promising results may indicate that our modeling strategy of applying probability calibration to ensemble learning is successful.

## Background

Diffuse large B-cell lymphoma (DLBCL), the most common subtype of B-cell non-Hodgkin lymphoma (NHL), accounts for 30–40% of all NHLs [1]. Due to its heterogeneity in clinical presentations and prognoses, DLBCL currently remains a significant clinical challenge [2, 3]. Although the application of rituximab has improved the overall survival rate, 30–50% of DLBCL patients remain sensitive to chemotherapy or relapse after remission and eventually die [4, 5]. In addition, under the influence of a chemotherapy regimen, a clinical staging, an immunologic expression or other factors, the prognoses of patients are markedly different [2, 6, 7]. Thus, according to clinicopathologic factors, we aim to predict the mortality hazard for patients with DLBCL at the individual level. Actually, clinicians need precise risk estimates for individuals to help them make optimal therapeutic decisions to achieve precision medicine [8]. Through early risk assessment, appropriate therapies may be initiated quickly and ultimately improve the clinical outcomes of individual cases [9, 10].

Instead of using a single model, we consider using ensemble learning to predict the 2-year mortality of patients with DLBCL. Ensemble learning can often achieve better performance than a single model by constructing and combining multiple models [11, 12]. These multiple models are also called individual models or base models. Dietterich [13] explained why the ensemble method works from statistical, computational and representational points of view. In addition, Kohavi [14] explained the reason for the success of the ensemble method from bias-variance decomposition. Due to its superior performance, ensemble learning has been applied in many fields. Wang Yuanchao [15] proposed a heterogeneous ensemble model composed of the random forest and XGBoost algorithms to classify pulsar candidates which achieved higher recall rate than other two algorithms. In [16], an ensemble of deep belief networks with different parameters was introduced into regression and time series forecasting. Several datasets were used for evaluation and eventually the ensemble-based model achieved better performance compared with other methods. Ensemble learning is also used to assist physicians in decision-making such as [17] which developed an ensemble model of deep belief networks for the early diagnosis of the Alzheimer’s Disease.

To achieve high performance, the base model in ensemble learning should have good accuracy and diversity [11, 12]. Diversity is key to ensemble learning, and there are three main ways to achieve diversity: data diversity, parameter diversity and structural diversity methods [12]. The data diversity method uses multiple datasets generated from the original dataset to train different base models. Since the multiple datasets are different from each other, the trained base models generate diverse outputs. The parameter diversity method trains different base models using different parameter sets. Even with the same algorithm or the same training data, the outputs from base models may vary with different parameters. The structural diversity method uses different algorithms to generate different base models and such ensemble models are also called heterogeneous ensembles. In our work, we consider five different algorithms for heterogeneous ensemble prediction, including the naïve Bayes (NB), logistic regression (logit), random forest (RF), support vector machine (SVM) and feedforward neural network (FNN) algorithms. However, some evidence [18–23] suggests that a few classifiers, such as the NB, RF and SVM classifiers, cannot generate precise probability estimates, although they achieve good classification performance in many real-world problems. Therefore, a different model structure is proposed to achieve both high accuracy and high diversity for the base models. That is, before aggregating the outputs from various base models, we first correct the poorly calibrated base models by using probability calibration methods. Probability calibration methods try to find a calibration function that maps the initial outputs of classifiers into more accurate posterior probabilities [18]. Many methods, such as the popular Platt scaling (Platt) and isotonic regression (IsoReg) approaches, have been proposed in this field. However, they all have their own preconditions. For example, Platt is effective when the output of the classifier is sigmoid-shaped, and IsoReg is prone to overfitting when the sample size is small. Thus, we calibrate the base model with shape-restricted polynomial regression (RPR), which is a flexible and powerful calibration method that is not constrained by a specific classifier [24]. By calibrating the base models first and then aggregating them, we hope to achieve strong ensemble performance.

Our research has three characteristics. First, this paper focuses not on the prediction of categories, but on membership probability. Second, a different model structure is proposed that applies probability calibration to ensemble learning. Third, both discrimination and calibration are considered in the model comparison.

## Methods

### Data sources and predictive variables

The data used in this study were derived from Shanxi Cancer Hospital, China. We assessed 406 patients diagnosed with DLBCL between April 2010 and May 2017, 116 of whom died within 2 years after treatment. We collected 17 variables for each patient from electronic medical records. See Table 1 for the names and groupings of each variable.

**Table 1:** The features and groupings of 406 patients with DLBCL

Features	Instances (N)
Age	≤ 60 (219), > 60 (187)
Gender	Male (209), Female (197)
Family history of cancer	No (369), Yes (37)
Stage	I, II (176), III, IV (230)
IPI	Low (195), Low-intermediate (79), High-intermediate (83), High (49)
KPS	≥ 80 (335), < 80 (71)
WBC	Low (80), Normal (297), High (29)
LDH	Normal (312), High (94)
*β*_2_-MG	Normal (296), High (110)
ESR	Normal (259), High (147)
Therapy	Chemotherapy alone (347), Chemotherapy and Radiotherapy (59)
GCB	Yes (243), No (163)
CD10	Negative (303), Positive (103)
Bcl-6	Negative (59), Positive (347)
MUM-1	Negative (222), Positive (184)
Ki-67	< 90 (303), ≥ 90 (103)
Rituximab	Not use (241), Use (165)

The figures in brackets represent the number of patients of this group.

*IPI* International prognostic index, *KPS* Karnofsky performance status, *WBC* White blood cell, *LDH* Lactate dehydrogenase, *β*_2_-MG *β*_2_- microglobulin, *ESR* Erythrocyte sedimentation rate, *GCB* Germinal center B-cell-like lymphoma; CD10, Bcl-6, MUM-1 and, Ki-67 are immunohistochemical indicators

We applied three methods to select the predictive variables, including the Cox model, the logistic model and the random forest algorithm. The Cox model uses outcomes and survival times as dependent variables to analyze the influencing factors of events. The results produced by the Cox model may not be reliable when the number of positive events is less than 10 times the number of covariates [25, 26]. Therefore, we first analyzed each variable by using the univariate Cox model. Then, the variables (including age, gender, stage, IPI, KPS, LDH, *β*_2_-MG, rituximab, and Bcl-6) that showed a univariate relationship (*P* <  0.1) with death were included in the multivariate Cox model. In addition to these 9 variables, we also added Ki-67 to the model. Ki-67 is a nuclear protein that can be used as a biomarker for cell proliferation, and its expression is widely used to evaluate the prognoses of many kinds of cancers, including lymphoma. A meta-analysis suggested that Ki-67 expression is associated with the prognoses of lymphoma patients. Subgroup analysis showed that high Ki-67 expression is highly associated with a poor overall survival rate for DLBCL [27]. Another study also suggested that patients with high Ki-67 expression have poor prognoses with standard first-line therapy [28]. Therefore, Ki-67 was also included in the multivariate Cox model in our study, although it was not significant in the univariate Cox analysis. Thus, these 10 variables were entered into the multivariate Cox model, and 8 variables remained (*P* <  0.1). The results of the multivariate Cox analysis are displayed in Table 2.

**Table 2:** The variables selected by the logit and Cox models when the threshold is 0.1

Variables	Groupings	Cox model		Logit model	
		Coefficient	*P*-value	Coefficient	*P*-value
Age	≤ 60	Reference	Reference	Reference	Reference
	>60	0.362	0.091	0.488	0.098
Gender	Male	Reference	Reference	Reference	Reference
	Female	−0.405	0.037	− 0.509	0.061
Stage	I, II	Reference	Reference	Reference	Reference
	III, IV	0.641	0.046	0.961	0.020
IPI	Low	Reference	Reference	Reference	Reference
	Low-intermediate	0.795	0.021	0.946	0.028
	High-intermediate	0.955	0.010	0.909	0.054
	High	0.726	0.100	0.807	0.160
KPS	≥ 80	Reference	Reference	Reference	Reference
	<80	0.877	< 0.001	1.350	< 0.001
LDH	Normal	Reference	Reference	Reference	Reference
	High	0.401	0.060	0.597	0.064
*β*_2_-MG	Normal	Reference	Reference	Reference	Reference
	High	0.395	0.061	0.562	0.066
Rituximab	Not use	Reference	Reference	Reference	Reference
	Use	−0.730	0.001	−0.931	0.002

*IPI* International prognostic index, *KPS* Karnofsky performance status, *LDH* Lactate dehydrogenase, *β*_2_-MG *β*_2_- microglobulin

**Fig. 1:**
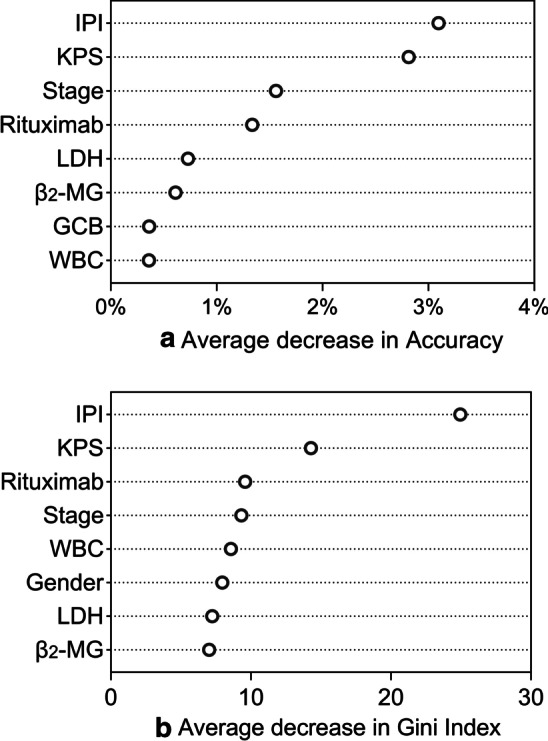
The ranking of variable importance (only showed top eight variables)

We also analyzed all the collected variables using the logit and RF approaches. The variables selected (*P* <  0.1) by the logit model were consistent with those selected by the Cox model (see Table 2). If the threshold was 0.05, the Cox model selected 5 variables (gender, stage, IPI, KPS, and rituximab), while logit selected 4 variables (stage, IPI, KPS, and rituximab). The only difference is that the Cox model contains “gender” and the logit model does not. Figure 1 shows the ranking of variable importance obtained by the RF algorithm. The ranking showed that stage, IPI, KPS, and rituximab were the four most important variables regardless of which importance metric was used. Moreover, we found that the ranking based on the Gini index (Fig. 1b) also included “gender” in the top eight variables.

According to the results of these three methods, we first used 0.05 as the threshold and took the union of the variables selected by the Cox and logit models as predictive variables (including gender, stage, IPI, KPS, and rituximab). Based on these 5 variables, we pretrained all the base models with 100 iterations. Then, we further added the remaining 3 variables (age, LDH, and *β*_2_-MG) to each base model. Although each base model at this point contained all 8 variables selected by the Cox and logit models when the threshold was 0.1, the predictive performance was not significantly improved. Thus, we excluded these 3 variables and only used gender, stage, IPI, KPS, and rituximab for prediction.

### Model structure

**Fig. 2:**
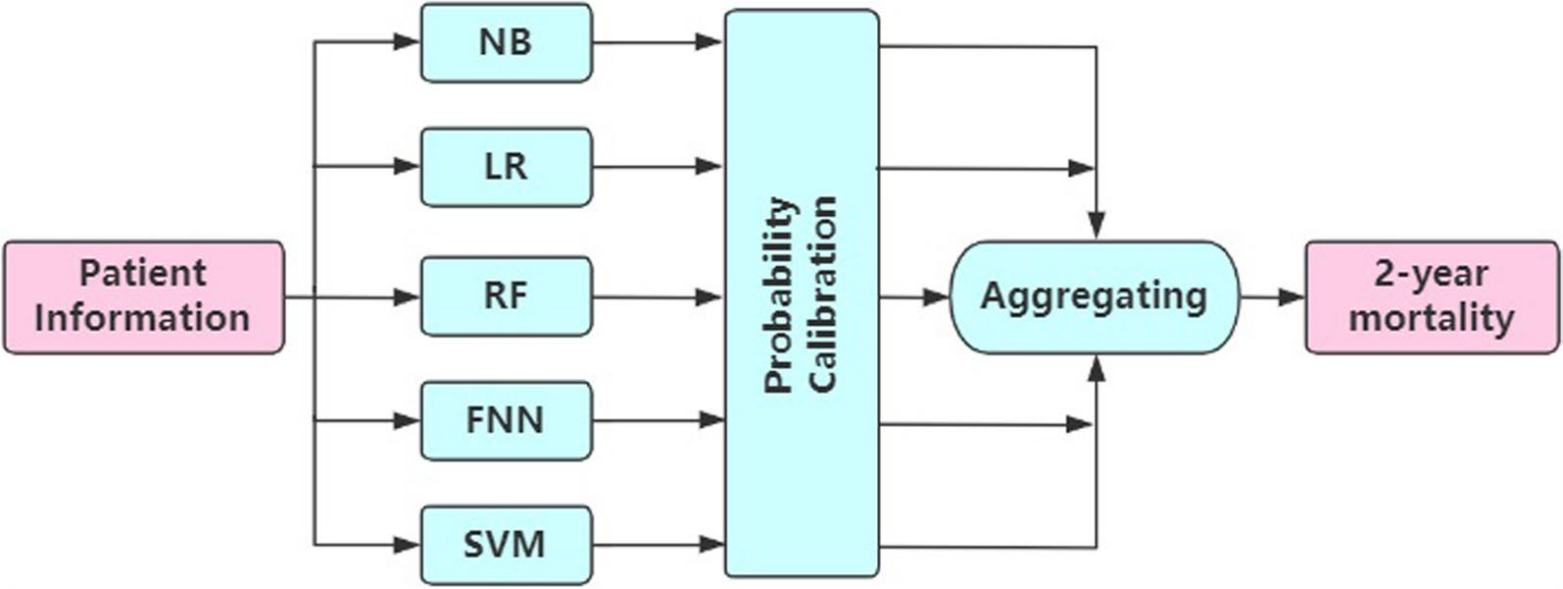
Schematic diagram of the model structure

As Fig. 2 shows, there are three main components of the model.

The first part is the construction of the base models. We used five common classifiers with good classification performances on many real-world problems as the base models, including the NB, Logit, RF, SVM, and FNN models. NB, which calculates the posterior probability that an observation belongs to each class based on Bayes’ theorem, classifies an observation as a member of the class for which the posterior probability is the largest. Logit is a generalized linear model used to solve classification problems. Since the model takes the logistic function as the link function, the posterior probability can be generated. The RF algorithm builds a number of decision trees based on bootstrapped training sets for prediction. In our RF, we used the voting ratio of all decision trees as the initial probability estimate. SVMs attempt to find an optimal separating hyperplane to classify observations into different classes. The sign of the output determines the class of the sample, and the magnitude of the output can be used as a measure of predictive confidence, since examples far from the hyperplane are more likely to be classified correctly than examples close to the hyperplane. The FNN, in which the neurons in each layer are fully connected to the neurons in the next layer and there is no loop in the network, is a common neural network structure. In our study, we built a 3-layer network, and the hidden layer included 500 units. We designed our network with a large number of hidden nodes because several studies suggest that a neural network with excess capacity generalizes better than a simple network when trained with back propagation and early stopping [29–31]. Other studies have also shown that a feedforward neural network can fit any continuous function of arbitrary complexity, and it only needs a single hidden layer containing enough units [32, 33].

The second part is the probability calibration of the base models. We selected RPR to calibrate the base models since it is a flexible and powerful method that is not constrained by a specific classifier [24]. We also applied two other methods (Platt, IsoReg) to investigate whether RPR was the best.

RPR calibrates initial probability using polynomial regression in which calibration function *f* has the following form [24]:

$$f\left( s \right) = ~{a_0} + {a_1}s + {a_2}{s^2} + \cdots + {a_k}{s^k} = \mathop \sum \limits_{l = 0}^k {a_l}{s^l}$$

where *s* is the initial probability of an observation. The solution is the following optimization problem:

$$\mathop {\min }\limits_{a \in {R^{k + 1}}} \frac{1}{N}\mathop \sum \limits_{n = 1}^N ~{\left[ {\mathop \sum \limits_{l = 0}^k {a_l}s_n^l - {y_n}} \right]^2}$$

a $$s.t. \,\, \mathop \sum \limits_{l = 0}^k {a_l}{^l} \ge 0, \quad \mathop \sum \limits_{l = 0}^k {a_l}{\bar s^l} \le 1$$

b $$\mathop \sum \limits_{l = 1}^k {a_l}l{s^{l - 1}} \ge 0, \quad \forall s\in \left[\ \underset{\_}{s},\overline{s}\ \right]$$

c $$\mathop \sum \limits_{l = 0}^k \left| {{a_l}} \right| \le \lambda$$

With constraint (a) all corrected probabilities are guaranteed to fall in the interval between 0 and 1. Constraint (b), which derives from the differentiability of *f*(*s*), ensures the monotonicity of the polynomial. In constraint (c), a *l*_1_-norm of ***a*** is used to prevent the polynomial from overfitting.

Platt is a parametric method which uses the sigmoid function as a calibration function [19]:

$$f(s)=\frac{1}{1+\exp \left( As+B \right)}$$

where A and B are estimated using the maximum likelihood estimation on the training set (*s*_*i*_, *y*_*i*_).

IsoReg is a nonparametric method which attempts to find an isotonic (i.e. non-decreasing) function that satisfies the following objective [21]:

$$Min~\frac{1}{N}~\mathop \sum \limits_{i = 1}^N ~{\left[ {~f\left( {{y_i}} \right) - ~{y_i}~} \right]^2} \quad s.t. \,\ {f_1} \le {f_2} \le \cdots \le {f_N}$$

where *y*_*i*_ = [ *y*_1_, *y*_2_, *y*_3_, …, *y*_*N*_] is the label sequence of all samples that has been sorted by their initial probabilities. A well-known method called pair adjacent violators (PAV) algorithm is used to estimate the isotonic function [34].

The third part is the combination of the base models. We used three methods (simple averaging, weighted averaging, and stacking) to combine the above 5 base models. Stacking or stacked generalization, which takes the outputs of the base models as its inputs, uses another machine learning algorithm (also called a meta-learner) to estimate the weight of each base model [35]. In our research, logit was used as a meta-learner. Since the outputs of all base models are numeric in our study, we also used simple averaging and weighted averaging to combine these base models and compared the results with those obtained by stacking. In weighted averaging, the weight of each base model was set to the reciprocal of the expected calibration error (ECE) and maximum calibration error (MCE). We first combined 5 uncalibrated base models (NB, logit, RF, SVM, FNN) by using these three methods. Then, according to our modeling strategy, we used the same methods to combine the calibrated base models (NB-RPR, logit, RF-RPR, SVM-RPR, FNN). We did not calibrate logit and FNN models because they could already generate accurate probability estimates, and their performances after calibration were not significantly improved.

### Model construction and evaluation

We combined the hold-out test with 3-fold cross-validation to complete the model construction and evaluation processes. To reduce statistical variability, all experiments were repeated 300 times. In each dataset, we randomly sampled 80% of the data (320 samples) as the training set and the residual 20% of the data (86 samples) as the testing set. Stratified sampling was used for each division to ensure the consistency of the data distribution across 300 experiments. In 3-fold cross-validation, the data were divided into 3 mutually exclusive subsets that were approximately equal in size. In each iteration, 2 of the 3 subsets were used as the training set, and the remaining subset was used as the validation set. The final evaluation depended on the average performance of the model on the 3 validation sets.

Amid the construction of the 5 base models, we first performed 3-fold cross-validation within the training set to find the optimal hyperparameters of the RF, SVM, and FNN models. Then, we used all training data to train these three models with the optimal hyperparameters, and assessed them within the testing set. For the RF algorithm, the number of candidate variables at each split was set to 2 and 3, and the choices for the number of trees were {500, 600, …, 1500}. For the SVM, the kernel function was selected from linear and Gaussian. The choices for the parameters C and Gamma were ${\left\{{10}^i\right\}}_{i=-4}^4$. In addition, we used all training data to fit the NB and logit models and assessed them within the testing set. Although they do not include hyperparameters, we also performed 3-fold cross-validation for them within the training set. For each base model, we finally extracted all the predictive values in the 3 validation sets as the training set of the calibration function.

Amid the probability calibration of the 5 base models, we first used 3-fold cross-validation within the calibration training set to determine the *k* and *λ* parameters of RPR. Then, we used the entire calibration training set to train the Platt, IsoReg, and RPR models with the optimal *k* and *λ* parameters. Finally, we calibrated the predictive values of the 5 base models in the testing set by using the trained Platt, IsoReg, and RPR models and then evaluated them. For the RPR, the choices for the degree *k* were {4, 5, 6, …, 20} and the choices for *λ* were ${\left\{{4}^i\right\}}_{i=-1}^5$.

Amid the combination of the 5 base models, the weight of each base model was set to 0.2 for simple averaging. For weighted averaging, the weight of each base model was set to the reciprocal of its ECE and MCE in the training data. For stacking, the outputs from the 5 base models were used as the inputs of the meta-learner (the logit model, in our study). To avoid overfitting, we used the union of the predictions of the base models on validation sets to train the meta-learner. Finally, we used the weights learned by stacking to combine the 5 base models.

Both discrimination and calibration were considered in the model evaluation. Discrimination and calibration are indispensable properties for assessing the accuracy of risk prediction models [36]. Discrimination is the ability to distinguish between patients who will have an event and those who will not. Calibration measures the consistency between the predicted probability and the true (observed) probability of patients at different risk strata. Although providing accurate probability estimates is our purpose, there is no need to further assess the calibration when the model has poor discrimination [36]. Therefore, we used the AUC to measure discrimination. The H-L test, ECE and MCE were used to evaluate the calibration of the models.

The H-L test is used to evaluate whether the difference between the predicted probability and the true probability is caused by chance [37]. The ECE and MCE are two measures related to the reliability diagram [38]. In computing these, the predictions are first sorted and then partitioned into *k* bins of equal size. For each bin, the predicted probability is the mean of the predictions in this bin, and the true (observed) probability is the proportion of positive observations in this bin. The ECE and MCE calculate the average prediction error and maximum prediction error over these bins, respectively:

$${\text{ECE}} = \mathop \sum \limits_{i = 1}^k \left| {{p_i} - {o_i}} \right|/k$$

$${\text{MCE}} = \max \left( {\left| {{p_i} - {o_i}} \right|} \right), \quad i = 1,2, \ldots ,k$$

where *p*_*i*_ and *o*_*i*_ are the predicted probability and the observed probability in the *i*-th bin, respectively. The lower the values of the ECE and MCE are, the lower the calibration error of the prediction.

The NB, logit, RF, and SVM models were implemented in the R 3.6 using the “e1071”, “glm”, “randomForest” and “e1071” packages. The FNN and RPR models were performed with Keras and CVXPY in the Python 3.6 [39, 40].

## Results

To reduce the variability caused by the data partition, the hold-out test was executed 300 times. The final evaluation was based on the average results of these 300 iterations.

### Performances of the 5 base models before and after calibration

We calibrated the NB, Logit, RF, SVM, and FNN models using RPR and compared the results with those of the Platt and IsoReg models. The performances of the 5 base models before and after calibration are shown in Table 3. The main features are summarized as follows:

**Table 3:** Performance of the 5 base models before and after calibration

	AUC	ECE	MCE	*P*
NB	**0.804 (0.773–0.839)**	14.206 (11.646–16.880)	38.900 (31.675–46.575)	< 0.001(< 0.001- < 0.001)
NB-Platt	**0.804 (0.773–0.839)**	9.966 (8.216–11.942)	**23.500 (19.100–29.925)**	0.250 (0.069–0.427)
NB-IsoReg	0.749 (0.686–0.792)	12.027 (8.577–15.908)	37.450 (25.000–53.550)	0.009(< 0.001–0.275)
NB-RPR	0.794 (0.767–0.830)	**9.514 (7.761–11.503)**	23.800 (17.900–30.625)	0.257 (0.072–0.536)
Logit	**0.803 (0.773–0.835)**	**9.517 (7.909–11.415)**	**24.400 (18.875–31.900)**	0.226 (0.055–0.486)
Logit-Platt	**0.803 (0.773–0.835)**	10.475 (8.588–12.490)	25.200 (19.450–33.200)	0.185 (0.057–0.409)
Logit-IsoReg	0.752 (0.697–0.794)	10.897 (8.234–15.700)	33.350 (21.720–50.820)	0.026(< 0.001–0.342)
Logit-RPR	0.801 (0.771–0.832)	9.784 (8.266–11.712)	24.600 (19.050–31.025)	0.244 (0.093–0.479)
RF	**0.800 (0.769–0.828)**	13.569 (11.453–15.771)	36.000 (30.580–41.630)	< 0.001(< 0.001- < 0.001)
RF-Platt	**0.800 (0.769–0.828)**	12.122 (9.999–14.068)	28.700 (22.500–35.800)	0.101 (0.022–0.284)
RF-IsoReg	0.777 (0.748–0.811)	**8.871 (6.431–11.491)**	28.600 (19.575–41.075)	0.185 (0.005–0.606)
RF-RPR	0.788 (0.753–0.816)	10.070 (7.989–11.932)	**26.550 (20.200–33.275)**	0.198 (0.042–0.464)
SVM	**0.792 (0.762–0.821)**	13.225 (11.390–15.112)	32.100 (25.420–39.970)	0.014 (0.001–0.102)
SVM-Platt	**0.792 (0.762–0.821)**	11.514 (9.373–13.996)	27.100 (21.650–34.170)	0.133 (0.026–0.352)
SVM-IsoReg	0.743 (0.676–0.784)	11.744 (8.644–15.926)	31.350 (21.975–47.525)	0.034(< 0.001–0.331)
SVM-RPR	0.788 (0.751–0.816)	**10.893 (8.880–13.453)**	**26.300 (20.300–34.320)**	0.140 (0.025–0.418)
FNN	**0.813 (0.780–0.845)**	**9.211 (7.075–10.391)**	**23.500 (17.675–28.925)**	0.329 (0.113–0.585)
FNN-Platt	**0.813 (0.780–0.845)**	9.990 (7.925–11.745)	23.600 (17.975–30.025)	0.243 (0.074–0.496)
FNN-IsoReg	0.731 (0.663–0.776)	14.090 (9.810–19.200)	46.000 (28.400–63.400)	< 0.001(< 0.001–0.079)
FNN-RPR	0.809 (0.776–0.838)	9.910 (7.540–11.020)	**23.500 (17.600–28.900)**	0.287 (0.103–0.531)

In each cell M (P_25_ - P_75_): M is the median, P_25_ is the 25th percentile and P_75_ is the 75th percentile of 300 tests. For each individual model, the best performance in each column is in bold and the secondary best performance in each column is underlined

*NB* Naïve Bayes, *Logit* Logistic regression, *RF* Random forest, *SVM* Support vector machine, *FNN* Feedforward neural network, *Platt* Platt scaling, *IsoReg* Isotonic regression, *RPR* Shape-restricted polynomial regression. “-Platt”, “-IsoReg” and “-RPR” represent performing probability calibration using corresponding method

**Fig. 3:**
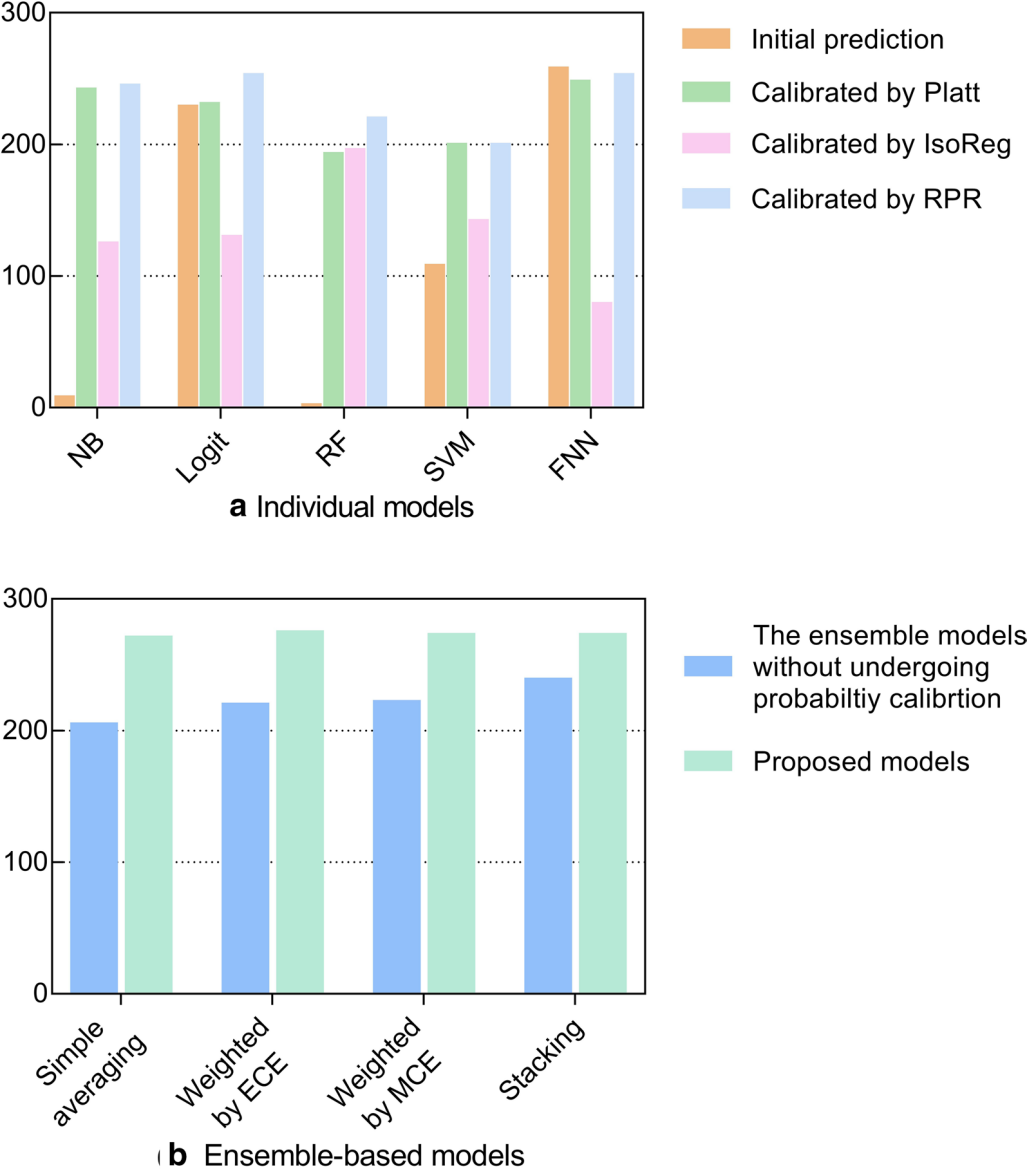
The frequency of achieving good calibration performance in 300 experiments

The AUCs of the 5 uncalibrated base models were all greater than 0.75, so they were considered as having good discrimination. Except for that of the SVM, the AUCs of the other 4 models were all greater than 0.8. In terms of calibration performance, the FNN had the lowest calibration error (ECE = 9.211, MCE = 23.500). The logit could also generate accurate probability estimates (ECE = 9.517, MCE = 24.400). According to the H-L test, these two models achieved good calibration (*P* > 0.05) 228 and 257 times out of 300 experiments. See Fig. 3a. By comparison, the NB, RF and SVM models had large prediction errors and they achieved good calibration only 7, 1, and 107 times out of 300 experiments, respectively.

After probability calibration was completed, the errors of the NB, RF and SVM models decreased significantly, and their frequencies of generating accurate probability estimates increased to 244, 219, and 196, respectively. However, regardless of which calibration method was used, the calibration error of the logit and FNN models were not further decreased. Out of the 3 evaluated probability calibration methods, RPR achieved the best correction for the SVM (ECE = 10.893, MCE = 26.300). IsoReg only achieved a good correction effect for the RF, while NB-IsoReg and SVM-IsoReg still had large calibration errors. According to Fig. 3a, NB-RPR, RF-RPR and SVM-RPR had higher frequencies of achieving good calibration in 300 experiments than the Platt and IsoReg models.

### Using 3 methods to combine the base models

First, we combined the NB, Logit, RF, SVM, and FNN models by simple averaging, weighted averaging using the ECE, weighted averaging using the MCE, and stacking. The results are presented in Table 4. Of all the models, the FNN had the best performance in terms of both discrimination and calibration (AUC = 0.813, ECE = 9.211, MCE = 23.500). Compared with the NB, RF and SVM models, the calibration errors of the 4 ensemble models decreased significantly.

**Table 4:** Performance of the ensemble model without undergoing probability calibration

	AUC	ECE	MCE	*P*
NB	0.804 (0.773–0.839)	14.206 (11.646–16.880)	38.900 (31.675–46.575)	< 0.001(< 0.001- < 0.001)
Logit	0.803 (0.773–0.835)	9.517 (7.909–11.415)	24.400 (18.875–31.900)	0.226 (0.055–0.486)
RF	0.800 (0.769–0.828)	13.569 (11.453–15.771)	36.000 (30.580–41.630)	< 0.001(< 0.001- < 0.001)
SVM	0.792 (0.762–0.821)	13.225 (11.390–15.112)	32.100 (25.420–39.970)	0.014 (0.001–0.102)
FNN	**0.813 (0.780–0.845)**	**9.211 (7.075–10.391)**	**23.500 (17.675–28.925)**	0.329 (0.113–0.585)
SA-EN	0.812 (0.778–0.843)	9.695 (7.968–11.699)	26.100 (19.600–32.650)	0.130 (0.025–0.332)
ECE-EN	**0.813 (0.778–0.844)**	9.228 (7.382–11.307)	24.500 (18.750–30.450)	0.186 (0.040–0.458)
MCE-EN	0.812 (0.777–0.843)	9.317 (7.456–11.156)	24.200 (18.700–30.525)	0.204 (0.046–0.445)
Stacking-EN	0.806 (0.771–0.834)	9.866 (8.416–11.763)	24.850 (19.275–30.425)	0.225 (0.074–0.435)

SA-EN, ECE-EN, MCE-EN, and Stacking-EN represent the ensemble models obtained by combining NB, Logit, RF, SVM, and FNN models using simple averaging, weighted averaging by the ECE, weighted averaging by the MCE, and stacking method respectively

In each cell M (P_25_ - P_75_): M is the median, P_25_ is the 25th percentile and P_75_ is the 75th percentile of 300 performances. The best performance in each column is in bold. The secondary best performance in each column is underlined

*NB* Naïve Bayes, *Logit* Logistic regression, *RF* Random forest, *SVM* Support vector Machine, *FNN* Feedforward neural network

Then, we combined the NB-RPR, Logit, RF-RPR, SVM-RPR, and FNN models in the same ways. The results are presented in Table 5. Stacking-EN-C performed the best out of all the models (AUC = 0.820, ECE = 8.983, MCE = 21.265). The MCEs of the 4 ensemble models were all lower than those of any of the base models. Except for SA-EN-C, the remaining 3 ensemble models had lower ECEs than that of any base model.

**Table 5:** Performance of the ensemble model that underwent probability calibration

	AUC	ECE	MCE	P
NB-RPR	0.794 (0.767–0.830)	9.514 (7.761–11.503)	23.800 (17.900–30.625)	0.257 (0.072–0.536)
Logit	0.803 (0.773–0.835)	9.517 (7.909–11.415)	24.400 (18.875–31.900)	0.226 (0.055–0.486)
RF-RPR	0.788 (0.753–0.816)	10.070 (7.989–11.932)	26.550 (20.200–33.275)	0.198 (0.042–0.464)
SVM-RPR	0.788 (0.751–0.816)	10.893 (8.880–13.453)	26.300 (20.300–34.320)	0.140 (0.025–0.418)
FNN	0.813 (0.780–0.845)	9.211 (7.075–10.391)	23.500 (17.675–28.925)	0.329 (0.113–0.585)
SA-EN-C	0.811 (0.777–0.842)	9.295 (7.634–11.199)	23.300 (17.800–29.650)	0.314 (0.131–0.536)
ECE-EN-C	0.811 (0.779–0.842)	9.027 (7.532–10.801)	22.350 (17.600–29.300)	0.351 (0.149–0.572)
MCE-EN-C	0.812 (0.777–0.842)	9.159 (7.456–10.862)	22.300 (17.100–28.700)	0.345 (0.161–0.566)
Stacking-EN-C	**0.820 (0.791–0.857)**	**8.983 (6.698–10.533)**	**21.265 (14.880–27.800)**	0.350 (0.145–0.672)

SA-EN-C, ECE-EN-C, MCE-EN-C, and Stacking-EN-C represent the ensemble models obtained by combining NB-RPR, Logit, RF-RPR, SVM-RPR, and FNN using simple averaging, weighted averaging by the ECE, weighted averaging by the MCE, and stacking method respectively

In each cell M (P_25_ - P_75_): M is the median, P_25_ is the 25th percentile and P_75_ is the 75th percentile of 300 performances. The best performance in each column is in bold. The secondary best performance in each column is underlined

*NB-RPR* Naïve Bayes calibrated by shape-restricted polynomial regression, *Logit* Logistic regression, *RF-RPR* Random forest calibrated by shape-restricted polynomial regression, *SVM-RPR* Support vector machine calibrated by shape-restricted polynomial regression, *FNN* feedforward neural network

Last, we compared the 4 ensemble models (SA-EN-C, ECE-EN-C, MCE-EN-C, Stacking-EN-C) that underwent probability calibration and the 4 ensemble models (SA-EN, ECE-EN, MCE-EN, Stacking-EN) that did not. Regardless of which combination of methods was used, the 4 ensemble models that underwent probability calibration had lower calibration errors in terms of both the ECE and MCE than the ensemble models that did not undergo probability calibration. According to the H-L test, our 4 ensemble models had the highest frequencies of achieving good calibration (*P* > 0.05), which were 270, 274, 272 and 272 out of 300 experiments. By comparison, the frequencies were 204, 219, 221 and 238 for the 4 ensemble models that did not undergo probability calibration. See Fig. 3b for the results.

## Discussions

We predicted 2-year mortality in patients with DLBCL at the individual level. The proposed method that applied probability calibration to ensemble learning performed satisfactorily in terms of both discrimination and calibration.

We used 5 variables (gender, stage, IPI, KPS, and rituximab) to predict the 2-year mortality of patients with DLBCL. Most of the 5 variables have been proven to affect the prognoses of patients with DLBCL. The development of rituximab is a breakthrough in the treatment of DLBCL, and current research indicates that rituximab can improve the clinical outcomes for almost all DLBCL subgroups [41–44]. IPI is a recognized prognostic indicator of DLBCL, and high IPI values are associated with poor prognoses of patients [45, 46]. An advanced disease stage has been reported to be a poor prognostic factor of DLBCL, and it is associated with a low overall survival rate and a short disease-free survival time [46]. Although there is no research about the relationship between KPS and the prognoses of patients with DLBCL, the statuses of patients [47] affect treatment options and, perhaps, patient outcomes.

Although the discrimination abilities of the 5 base models are very similar, the differences in calibration are obvious. The logit and FNN models can accurately predict the 2-year mortality of DLBCL patients. However, the errors of the initial probabilities of the NB, RF and SVM models are large. These results are consistent with those of several reports [18–23]. The logit and FNN models return well-calibrated predictions because they directly optimize the log-loss of probability [23]. For the NB classifier, the output is often pushed to 0 or 1 because the required conditional independence assumption may not be valid in reality [21–23]. Inversely, the output of the RF algorithm is often pushed away form 0 and 1 because it is difficult to obtain identical predictions from all decision trees [18, 20, 23]. Furthermore, the SVM also pushes the prediction away from 0 and 1 and induces a sigmoid-shaped distortion. Although the magnitude of the output can be taken as a measure of confidence in the prediction, these values are often poorly calibrated [19, 23].

The Platt model is effective when the distortion of the prediction is sigmoid-shaped. In our study, the biased probabilities obtained by the NB, RF and SVM models achieved good correction effects by using the Platt model. IsoReg is a universal calibration method in which the only restriction is that the mapping function is isotonic (i.e. non-decreasing). However, the calibration errors of the NB and SVM models are still large after calibration by IsoReg. This may be caused by an overfitting effect resulting from scarce data. A report by Niculescu-Mizil suggested that IsoReg may not be suitable for calibration with a small sample size, especially when the sample size is less than 1000 [23]. By comparison, RPR is more flexible and powerful. Unlike the Platt model, RPR can calibrate any classifier since it has no constraints on the distribution of the initial output. Compared with IsoReg, RPR uses polynomial regression as a calibration function so that it is continuous over the entire interval. In addition, RPR strictly satisfies the monotonicity requirement and can be solved conveniently by optimization tools such as CVXPY [39, 40]. RPR can theoretically fit any calibration function as the polynomial degree increases. Therefore, RPR was used to correct poorly calibrated classifiers in our study. According to the H-L test, NB-RPR, RF-RPR and SVM-RPR had the highest frequencies of achieving good calibration in 300 experiments compared with the results of the Platt and IsoReg models. Although the MCE of NB-Platt was lower than that of NB-RPR and the ECE of RF-IsoReg was lower than that of RF-RPR, we believe that RPR achieved better calibration for the NB, RF and SVM models than the other calibration methods if we also consider the results of the H-L test.

Ensemble methods can obtain better performance than single models by combining multiple models, especially when the individual models are weak. Many theories in this field were initially proposed for weak models, such as the family of boosting models. Although aggregating weak models is sufficient for obtaining good performance in theory, models with good accuracy are often used as the base model in practice, especially when few base models are available. In our work, we used probability calibration to improve the accuracy of the base models’ probabilistic predictions to obtain strong ensemble performance. The results show that the calibration performance of the ensemble model was improved by calibrating the base models first. In terms of both the ECE and MCE, the calibration errors of our 4 ensemble models (SA-EN-C, ECE-EN-C, MCE-EN-C, Stacking-EN-C) were lower than those of the 4 ensemble models (SA-EN, ECE-EN, MCE-EN, Stacking-EN) that did not undergo probability calibration. According to the H-L test, our 4 ensemble models had the highest frequencies of achieving good calibration performance in 300 experiments, which were 270, 274, 272 and 272. However, they were 204, 219, 221 and 238 for the 4 ensemble models that did not undergo probability calibration. For all models, the stacking method that first calibrated the base model by RPR performed best in terms of both discrimination and calibration (AUC = 0.820, ECE = 8.983, MCE = 21.265). By contrast, the NB classifier generates probabilistic predictions with the highest error (ECE = 14.206, MCE = 38.900). Overall, our model, which applies probability calibration to ensemble learning, achieves the desired results.

Our research has limitations. First, the bootstrapping method may be more appropriate for the model evaluation of our study than the hold-out method. Since the sample size of this research is small, the model trained on the bootstrapped dataset, which has the same sample size with the training data, may be has lower estimation error. Second, it may be possible to improve the maximum calibration error in our study. The probability after calibration may not change significantly, as the calibration function has to ensure global monotonicity over the entire range. This means that the calibration error is largely influenced by misclassified samples. In the future, we will collect more feature information about DLBCL patients to improve the discrimination ability of the model, which may further increase the accuracy of its probabilistic predictions. Third, we only selected five common classifiers as the base models in our work. The impact of increasing or decreasing the number of base models or performing selective ensemble learning on the final prediction can be further investigated. Fourth, this research was based on the data provided by a certain hospital. Therefore, an external validation is required to evaluate the generalizability of the model.

## Conclusions

In conclusion, we predicted the 2-year mortality of patients with DLBCL at the individual level. The proposed method performs satisfactorily and has the potential to help clinicians improve the outcomes of individuals by providing accurate risk predictions. Furthermore, these promising results may indicate that our modeling strategy of applying probability calibration to ensemble learning is successful.

## Data Availability

The dataset generated and analyzed during the current study are not publicly available due to subsequent studies have not been completed but are available from the corresponding author on reasonable request. In addition, we agreed to share all the code in this study which has been written into separated file. Therefore, other interested researchers can easily implement our model.

## Abbreviations

DLBCL: Diffuse Large B-cell Lymphoma
NB: Naïve Bayes
Logit: Logistic Regression
RF: Random Forest
SVM: Support Vector Machines
FNN: Feedforward Neural Network
Platt: Platt Scaling
IsoReg: Isotonic Regression
RPR: Shape-restricted Polynomial Regression
IPI: International Prognostic Index
KPS: Karnofsky Performance Status
AUC: Area Under the ROC Curve
H-L: Hosmer-Lemeshow
ECE: Expected Calibration Error
MCE: Maximum Calibration Error.

## References

[1] Jemal A, Siegel R, Xu JQ (2013). Cancer statistics. CA Cancer J Clin.

[2] Roschewski M, Staudt LM, Wilson WH (2014). Diffuse large B-cell lymphoma—treatment approaches in the molecular era. Nat Rev Clin Oncol.

[3] Pasqualucci L, Dalla-Favera R (2018). Genetics of diffuse large B-cell lymphoma. Blood.

[4] Martelli M, Ferreri AJM, Agostinelli C (2013). Diffuse large B-cell lymphoma. Crit Rev Oncol Hematol.

[5] Tilly H, Vitolo U, Walewski J (2012). Diffuse large B-cell lymphoma (DLBCL): ESMO Clinical Practice Guidelines for diagnosis, treatment and follow-up. Ann Oncol.

[6] Horn H, Ziepert M, Wartenberg M (2015). Different biological risk factors in young poor-prognosis and elderly patients with diffuse large B-cell lymphoma. Leukemia.

[7] Morrison VA, Hamlin P, Soubeyran P (2015). Diffuse large B-cell lymphoma in the elderly: impact of prognosis, comorbidities, geriatric assessment, and supportive care on clinical practice. An International Society of Geriatric Oncology (SIOG) expert position paper. J Geriatr Oncol.

[8] Jameson JL, Longo DL (2015). Precision medicine — personalized, problematic, and promising. N Engl J Med.

[9] Stenberg E, Cao Y, Szabo E (2018). Risk prediction model for severe postoperative complication in bariatric surgery. Obes Surg.

[10] Degnim AC, Winham SJ, Frank RD (2018). Model for predicting breast cancer risk in women with atypical hyperplasia. J Clin Oncol.

[11] Zhou ZH (2016). Ensemble learning. Maching learning.

[12] Ren Y, Zhang L, Suganthan PN (2016). Ensemble classification and regression-recent developments, applications and future directions. IEEE Comput Intell Mag.

[13] Dietterich T.G. (2000) Ensemble methods in machine learning. In: Multiple classifier systems. MCS 2000. Lecture notes in computer science, vol 1857. Berlin, Heidelberg: Springer. 10.1007/3-540-45014-9_1.

[14] Kohavi R, Wolpert DH. Bias plus variance decomposition for zero-one loss functions. In: ICML'96: Proceedings of the thirteenth international conference on international conference on machine learning. 1996. p. 275–283.

[15] Wang Y, Pan Z, Zheng J (2019). A hybrid ensemble method for pulsar candidate classification. Astrophysics Space Sci.

[16] Qiu X, Zhang L, Ren Y (2015). Ensemble deep learning for regression and time series forecasting. Computational intelligence in ensemble learning.

[17] Ortiz A, Munilla J, Górriz JM (2016). Ensembles of deep learning architectures for the early diagnosis of the Alzheimer’s disease. Int J Neural Syst.

[18] Boström H (2008). Calibrating random forests. Seventh Int Conf Mach Learn Appl.

[19] Platt J (1999). Probabilistic outputs for support vector machines and comparisons to regularized likelihood methods. Adv Large Margin Classifiers.

[20] Boström H. Estimating class probabilities in random forests. In: Sixth international conference on machine learning and applications (ICMLA 2007), Cincinnati, OH. 2007. p. 211–216. 10.1109/ICMLA.2007.64.

[21] Zadrozny B, Elkan C (2002). Transforming classifier scores into accurate multiclass probability estimates. Proceedings of the Eighth ACM SIGKDD International Conference on Knowledge Discovery and Data Mining.

[22] Zadrozny B, Elkan C (2001). Obtaining calibrated probability estimates from decision trees and naive Bayesian classifiers. ICML.

[23] Niculescu-Mizil A, Caruana R (2005). Predicting good probabilities with supervised learning.

[24] Wang Y, Li L, Dang C (2019). Calibrating classification probabilities with shape-restricted polynomial regression. IEEE Trans Pattern Anal Mach Intell.

[25] Stone GW, Maehara A, Lansky AJ (2011). A prospective natural-history study of coronary atherosclerosis. N Engl J Med.

[26] Peduzzi P, Concato J, Feinstein AR (1995). Importance of events per independent variable in proportional hazards regression analysis II. Accuracy and precision of regression estimates. J Clin Epidemiol.

[27] He X, Chen Z, Fu T (2014). Ki-67 is a valuable prognostic predictor of lymphoma but its utility varies in lymphoma subtypes: evidence from a systematic meta-analysis. BMC Cancer.

[28] Song MK, Chung JS, Lee JJ (2015). High Ki-67 expression in involved bone marrow predicts worse clinical outcome in diffuse large B cell lymphoma patients treated with R-CHOP therapy. Int J Hematol.

[29] Weigend A (1993). On overfitting and the effective number of hidden units. Proc Connectionist Models Summer School.

[30] Caruana R, Lawrence S, Giles CL. Overfitting in neural nets: backpropagation, conjugate gradient, and early stopping. In: Advances in neural information processing systems. 2000. p. 402–408.

[31] Lawrence S, Giles CL, Tsoi AC. Lessons in neural network training: overfitting may be harder than expected. In: National conference on artificial intelligence. 1997. p. 540–545.

[32] Hornik K, Stinchcombe M, White H (1989). Multilayer feedforward networks are universal approximators. Neural Netw.

[33] Zhou ZH (2016). Neural networks. Maching learning.

[34] Ayer M, Brunk HD, Ewing GM (1955). An empirical distribution function for sampling with incomplete information. Ann Math Stat.

[35] Wolpert DH (1992). Stacked generalization. Neural Netw.

[36] Alba AC, Agoritsas T, Walsh M (2017). Discrimination and calibration of clinical prediction models: users’ guides to the medical literature. JAMA.

[37] Hosmer DW, Hosmer T, Le Cessie S (1997). A comparison of goodness-of-fit tests for the logistic regression model. Stat Med.

[38] Naeini MP, Cooper GF, Hauskrecht M (2015). Binary classifier calibration using a Bayesian non-parametric approach. Proceedings of the 2015 SIAM International Conference on Data Mining.

[39] Diamond S, Boyd S (2016). CVXPY: a Python-embedded modeling language for convex optimization. J Mach Learn Res.

[40] Agrawal A, Verschueren R, Diamond S (2018). A rewriting system for convex optimization problems. J Control Decis.

[41] Coiffier B, Lepage E, Brière J (2002). CHOP chemotherapy plus rituximab compared with CHOP alone in elderly patients with diffuse large-B-cell lymphoma. N Engl J Med.

[42] Pfreundschuh M, Trümper L, Osterborg A (2006). CHOP-like chemotherapy plus rituximab versus CHOP-like chemotherapy alone in young patients with good-prognosis diffuse large-B-cell lymphoma: a randomised controlled trial by the MabThera international trial (MInT) group. Lancet Oncol.

[43] Coiffier B, Thieblemont C, Van Den Neste E (2010). Long-term outcome of patients in the LNH-98.5 trial, the first randomized study comparing rituximab-CHOP to standard CHOP chemotherapy in DLBCL patients : a study by the Groupe d'Etudes des Lymphomes de l'Adulte. Blood.

[44] Fu K, Weisenburger DD, Choi WWL (2008). Addition of rituximab to standard chemotherapy improves the survival of both the germinal center B-cell-like and non-germinal center B-cell-like subtypes of diffuse large B-cell lymphoma. J Clin Oncol.

[45] Chinese Society of Hematology (2013). Guidelines for the diagnosis and treatment of diffuse large B-cell lymphoma in China (2013 edition). Chin J Hematol.

[46] Zhang A, Ohshima K, Sato K (2010). Prognostic clinicopathologic factors, including immunologic expression in diffuse large B-cell lymphomas. Pathol Int.

[47] Zelenetz A, Gordon L, Abramson J (2019). NCCN clinical practice guidelines in oncology: B-cell lymphomas, Version 3.2019. J Natl Compr Canc Netw..

